# The evaluation of inflammatory and immune composite markers for complications after deceased donor liver transplantation – a retrospective cohort study

**DOI:** 10.1080/07853890.2025.2536757

**Published:** 2025-07-24

**Authors:** Zhihong Zheng, Shuman Kuang, Zipei Wang, Jin Gu, Jichang Jiang, Zhou Yu, Lijin Zhao

**Affiliations:** Department of General Surgery, Digestive Disease Hospital, Affiliated Hospital of Zunyi Medical University, Zunyi City, Guizhou Province, China

**Keywords:** Liver transplantation, postoperative complications, composite markers, early allograft dysfunction, composite markers, liver transplantation, postoperative complications, predictive model, early allograft dysfunction

## Abstract

**Background:**

Inflammation-immune composite markers have been increasingly investigated in various diseases, but their value in the setting of deceased donor liver transplantation remains insufficiently investigate.

**Method:**

A retrospective cohort study was conducted on deceased donor liver transplant recipients at Zunyi Medical University. Univariate and multivariate logistic regression analyses identified independent risk factors for major complications. A nomogram was constructed based on these factors. Model performance was evaluated using ROC curve, calibration curve, decision curve analysis, and clinical impact curve.

**Results:**

Preoperative factors such as donor age, MLR, Anhepatic, Total Cholesterol, CIT, CAR, Lactate, and SII were identified as independent risk factors for postoperative complications with CD ≥ III but < IV, as well as CD ≥ IV complications. The Nomogram based on these results demonstrated good predictive ability and stability in the Bootstrap validation.

**Conclusion:**

Preoperative MLR and SII showed good predictive ability for postoperative complications graded as CD ≥ III but < IV, while CAR was predictive of CD ≥ IV complications and was associated with EAD and prolonged ICU stay. MLR was an independent risk factor for 90-day postoperative mortality. The nomogram of major outcomes requires further validation in multi-center studies.

## 1. Introduction

Liver transplantation (LT) is the most effective treatment for end-stage liver disease, acute liver failure(ALF), and malignant hepatic tumors [1–3]. Since the first LT was performed, this technique has undergone years of development. Advances in surgical techniques, the understanding of liver immunology, and the criteria for donor and recipient selection have significantly improved patient outcomes [4–6]. However, LT is still associated with severe complications, early patient mortality, and graft failure. Serious complications such as hepatic artery thrombosis (HAT) and primary nonfunction (PNF) can lead to early patient death and often require retransplantation for treatment [7–11]. The number and types of complications after LT are numerous and varied, including both graft-related and non-graft-related complications. These complications are difficult to define by any specific clinical feature and often impact patient prognosis [12,13]. In some cases, recipients may develop complications within a few days after transplantation, which can progress rapidly, leading to retransplantation or even death. Therefore, there is a strong rationale for the early identification of high-risk individuals for post-LT complications, as this would allow for earlier monitoring, as well as preventive and interventional measures in recipients at high risk of complications, ultimately saving clinical resources and improving patient outcomes. In this context, several novel prognostic scoring models have been proposed, such as the Model for End-Stage Liver Disease (MELD score) [14]. Survival Outcomes Following Liver Transplantation (SOFT) score [8], L-GrAFT score [15] MEAF score, and Early Allograft Failure Simplified Estimation (EASE) score [16]. Additionally, several predictive biomarkers for post-LT complications have been identified, including recipient age, serum creatinine levels, bilirubin, and donor and recipient BMI [17–20].

Recently, composite indices combining single inflammatory and immune markers from recipient serum have gained attention, such as the C-reactive protein to albumin ratio (CAR), neutrophil to lymphocyte ratio (NLR), platelet to lymphocyte ratio (PLR), and systemic inflammation index (SII), among others. These inflammation-immune composite indices can comprehensively consider various aspects of characteristics. Several studies have confirmed that they outperform traditional individual laboratory markers and other inflammation scores in predicting outcomes in various diseasesand populations [21–29]. However, in the context of LT, research on these indices has primarily focused on Living Donor Liver Transplantation (LDLT), with fewer studies conducted in Deceased Donor Liver Transplantation (DDLT). Currently, studies have reported that inflammation-immune composite indices in LDLT are significantly associated with Early Allograft Dysfunction (EAD), 1-year transplant failure rates, and post-transplant recurrence of hepatocellular carcinoma, and are also correlated with decreased survival rates [30–34]. However, studies have demonstrated that recipients of LDLT and DDLT exhibit different postoperative complication characteristics, with significant differences in the incidence rates of the same complications between the two groups [13,35]. Compared with LDLT, DDLT grafts are more likely to experience a more intense ischemia-reperfusion injury (IRI)-associated inflammatory cascade—such as heightened proinflammatory cytokine release and oxidative stress—due to several intrinsic donor-related factors, including unstable donor physiology, significantly longer cold ischemia time (typically three times longer than in LDLT), and a higher degree of hepatic steatosis [36]. These donor-related differences exacerbate the postoperative immune response and lead to elevated release of damage-associated molecular patterns (DAMPs). Previous studies have identified DDLT itself as an independent risk factor for post-transplant complications, including patient mortality and graft loss [37]. Therefore, it is necessary to conduct DDLT-specific investigations. To our knowledge, no study has systematically examined the predictive ability of such composite indices for early postoperative complications and various outcomes in DDLT recipients. Therefore, the aim of this study is to systematically investigate the predictive ability of these inflammation-immune composite indices for various outcomes in DDLT recipients, while also developing a Nomogram based on the risk factors for complications to assist clinical decision-making and provide insights for the development of future models.

## 2. Materials and methods

### 2.1. Study design and endpoint

**Figure 1.**
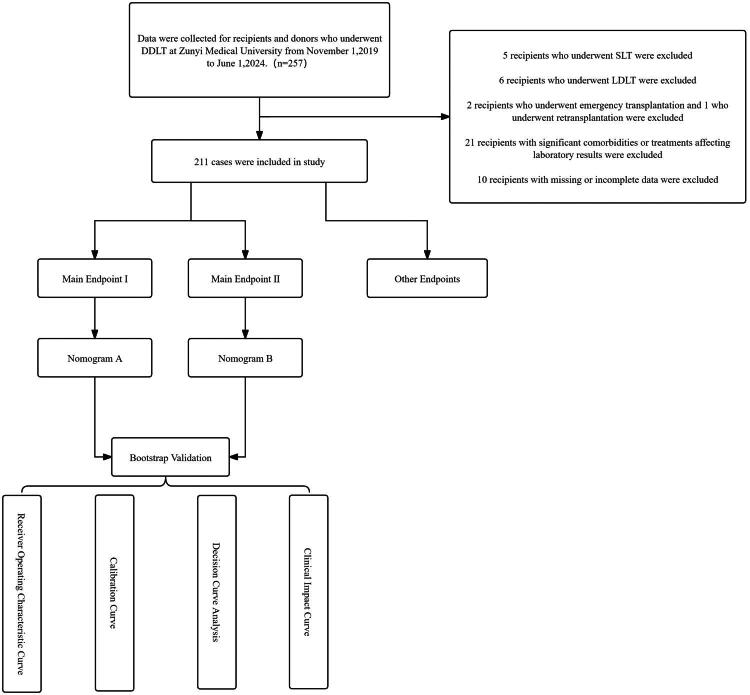
Flow chart of study.

A retrospective collection of baseline data, preoperative and intraoperative data, and 90-day postoperative follow-up data of liver transplant recipients and donors from Zunyi Medical University Affiliated Hospital, between November 1, 2019, and June 1, 2024, was conducted. The transplant procedures involved in this study were performed between November 2019 and June 2024. After applying the inclusion and exclusion criteria, a total of 211 cases were included in this study. According to the Clavien-Dindo classification of complications, complications requiring surgical intervention within 90 days after liver transplantation (Clavien-Dindo grade ≥ III but < IV) were defined as primary outcome I, while complications that could potentially threaten life (Clavien-Dindo grade ≥ IV) were defined as primary outcome II. The secondary outcomes of the study include: the Comprehensive Complication Index (CCI) calculated at 90 days post-LT; 90-day mortality rate; 90-day graft loss rate; postoperative Intensive Care Unit (ICU) length of stay; total hospitalization duration; Early Allograft Dysfunction (EAD) defined by the Olthoff criteria; and total hospitalization costs for the LT procedure (Figure 1).

### 2.2. Data collection

This study collected the following donor characteristic data for analysis: age, sex, blood type, weight, and BMI. The following recipient characteristic data were collected for analysis: age, sex, blood type, weight, BMI, preoperative MELD score, C-reactive protein, albumin, absolute neutrophil count, absolute lymphocyte count, absolute monocyte count, platelet count, liver transaminases (AST and ALT), creatinine, total bilirubin, total cholesterol, lactate, uric acid, PT, APTT, INR, total length of hospital stay, postoperative ICU stay duration, total hospitalization costs, and 90-day postoperative follow-up data. Surgical-related indicators collected include: warm ischemia time (WIT), cold ischemia time (CIT), anhepatic phase, graft weight, and the graft-to-recipient weight ratio (GRWR). The inflammation-immune composite indices used in this study were all calculated from the collected laboratory test results. CAR = C-reactive protein to albumin ratio; NLR = Neutrophil to Lymphocyte Ratio; MLR = Monocyte to Lymphocyte Ratio; PLR = Platelet to Lymphocyte Ratio; Albumin-Bilirubin Index (ALBI) = log10(Total Bilirubin (μmol/L)) × 0.66 + (Albumin (g/L)) × (−0.085); SII = Neutrophil count × Platelet count/Lymphocyte count. In this study, organ donors did not include vulnerable groups such as prisoners; all donors voluntarily donated after signing an informed consent form provided by family members. Between December 15, 2024, and December 31, 2024, laboratory test data of the cases and baseline data of the donors and recipients were collected through the hospital’s electronic medical record system and the China Liver Transplant Registry Center (www.cltr.org). Hematological data required for inflammatory and immune composite markers were obtained using the Sysmex XN9000-1 automated hematology analyzer (Sysmex Corporation, Japan) with matching reagents. Other biochemical parameters were measured using the Beckman Coulter AU5800 analyzer (Beckman Coulter, USA) and corresponding reagents. All tests were performed in the hospital’s clinical laboratory. The laboratory test data from the day before the recipient’s surgery were systematically collected, and in cases of multiple tests conducted on the same day, the most recent test data closest to the surgery time were selected.

### 2.3. Exclusion criteria

The cases that met the following exclusion criteria were excluded from the study:

- Receiving emergency transplantation/re-transplantation;
- Receiving split liver transplantation (SLT) or LDLT;
- Donor/recipient age < 18 years;
- Having significant comorbidities prior to LT that substantially affect preoperative laboratory results (e.g. severe infections requiring pre-transplant hospitalization, ascites requiring surgical or interventional intervention, preoperative renal replacement therapy, or receiving high-dose immunosuppressive or steroid therapy);
- :Recipients who were unable to complete routine standardized postoperative management for various reasons (e.g. immunosuppressive and anti-infective treatments);
- Missing/incomplete data or loss to follow-up.

### 2.4. Ethical statement

All transplant donors involved in this study were not from vulnerable populations, and all donors or their close relatives provided freely given written informed consent. The authors are responsible for all aspects of the work, ensuring that any issues related to the accuracy or integrity of any part of the work are properly investigated and resolved. The study was conducted in accordance with the Declaration of Helsinki (2013) and the Declaration of Istanbul (2018) The study was approved by the ethics board of Zunyi Medical University Affiliated Hospital (No. KLLY-2024-130), and individual consent for this retrospective analysis was waived. Throughout the entire organ donation process, no other forms of financial compensation, including hospitalization fees, were involved. The authors are accountable for all aspects of the work in ensuring that questions related to the accuracy or integrity of any part of the work are appropriately investigated and resolved.

### 2.5. Statistical analysis

Data analysis, nomogram creation, and image plotting in this study were conducted using SPSS (Version 29.0, Chicago, IL, USA) and R software (Version 4.4.2). The Kolmogorov-Smirnov test was used to assess the normality of the data. Categorical variables were expressed as frequencies (percentages), and continuous variables were expressed as mean ± standard deviation (SD) or median and interquartile ranges (IQRs). Group comparisons were performed using the following statistical methods based on the data type and distribution: Student’s t-test, Mann-Whitney U test, chi-square test, Fisher’s exact test, or Kruskal–Wallis H test. Spearman correlation analysis was used to evaluate the correlations between various inflammation-immune composite indices and secondary outcomes. Statistical significance was defined as a two-sided p-value < 0.05. Independent risk factors for binary outcomes were identified through univariate and multivariate binary logistic regression analysis, and a nomogram was plotted. The Bootstrap method was used when appropriate to balance sampling bias. The optimal cutoff values were determined by the Youden Index, which is calculated from the receiver operating characteristic (ROC) curve. The discriminative ability of the nomogram model was assessed using the concordance index (C-index) and validated using the Bootstrap method, along with a calibration curve. The calibration of the model was evaluated using the Hosmer-Lemeshow (HL) goodness-of-fit test. The value of the C-index corresponds to the area under the curve (AUC), which ranges from 0.5 to 1.0: 0.5 indicates a predictive ability similar to random chance, and 1.0 indicates perfect predictive ability.

## 3. Result

### 3.1. Patient characteristics

During the study period, 257 liver transplants were performed. After applying exclusion criteria—including 5 SLT, 7 LDLT, 3 emergency or re-transplantations, 21 recipients with comorbidities affecting laboratory results, and 10 cases with incomplete data—211 cases remained. Among them, 158 (74.9%) were male and 53 (25.1%) female. Median donor and recipient ages were 43 [18] and 50 [12] years, respectively. The leading indications for transplantation were hepatitis B virus-related cirrhosis (33.6%), hepatocellular carcinoma (HCC, 32.7%), and acute liver failure (ALF, 11.4%). Donor death was most commonly due to cerebrovascular accident (CVA, 62.1%), followed by severe trauma (23.7%) and poisoning (6.6%).

Main Endpoint I was defined as complications requiring surgical or interventional treatment within 90 days post-transplantation (CD grade ≥ III and < IV), with 101 cases (47.9%) observed. Main Endpoint II, defined as severe or fatal complications within 90 days (CD grade ≥ IV), occurred in 39 patients (18.5%). A total of 25 patients (11.8%) across both endpoint groups died within 90 days. The median CCI score was 43.4 [34.6]. EAD and graft loss within 90 days were reported in 52 (24.6%) and 11 (5.2%) patients, respectively. The median total hospital stay was 18 [10] days. Among the 211 patients, 162 (76.8%) did not require postoperative ICU admission, while 45 (21.3%) had ICU stays of more than one day. Of these, about 21 patients (10.0%) stayed for 3–5 days. The maximum ICU stay observed was 29 days, reported in two patients (0.9%). Detailed baseline characteristics of donors and recipients, as well as major outcomes and study variables, are presented in Table 1.

**Table 1.** Statistical characteristics of baseline characteristics of main endpoints.

Variables	All patients (*n* = 211)	CD ≥ III and < IV(*n* = 101)	CD > IV(*n* = 39)	P-value
Donor				
Age	42.97 ± 13.13	45.89 ± 12.01	43[17]	0.057
Weight	63.87 ± 8.90	63.78 ± 6.70	64.82 ± 10.42	0.889
BMI	23.31 ± 2.47	23.17 ± 2.54	23.72 ± 2.33	0.172
SEX				
Male	176(83.4)	83(82.2%)	35(89.74%)	0.498
Female	35(16.6)	18(17.82%)	4(10.25%)	
BloodType				
A	57(27.0%)	31(30.69%)	7(17.94%)	0.745
B	60(28.4%)	26(25.74%)	14(35.89%)	
AB	14(6.6%)	8(7.92%)	2(5.12%)	
O	80(37.9%)	36(35.64%)	16(41.02%)	
Cause Of Death				
CVA	131(62.1%)	66(65.34%)	18(46.15%)	0.707
Trauma	50(23.7%)	22(21.78%)	13(33.33%)	
Poison	14(6.6%)	5(4.95%)	4(10.25%)	
Ohters	16(7.5%)	8(7.92%)	4(10.25%)	
Recipient				
Age	48.25 ± 11.56	49.22 ± 9.44	46.82 ± 9.56	0.722
Weight	62.07 ± 13.22	62.33 ± 10.53	62.23 ± 12.75	0.794
BMI	23.35 ± 3.54	23.08 ± 2.99	23.55 ± 5.77	0.456
SEX				
Male	158(74.9%)	81(80.20%)	27(69.23%)	0.231
Female	53(25.1%)	20(19.80%)	12(30.76%)	
BloodType				
A	65(30.8%)	32(31.68%)	9(23.07%)	0.747
B	56(26.5%)	25(24.75%)	13(33.33%)	
AB	18(8.5%)	11(10.89%)	2(5.12%)	
O	72(34.1)	33(32.67%)	15(38.46%)	
Etiology				
Hepatitis B Virus-Related Cirrhosis	71(33.6%)	25(24.75%)	11(28.20%)	0.144
HCC	69(32.7%)	39(38.61%)	14(35.89%)	
Acute Liver Failure	9(4.3%)	7(6.93%)	1(2.56%)	
Others	62(29.4%)	30(29.70%)	13(33.33%)	

CVA: Cerebrovascular Accident;HCC: Hepatocellular Carcinoma; Normally distributed variables are presented as mean ± standard deviation, while non-normally distributed variables are presented as median [IQRs];Categorical variables are presented as frequency (percentage).

### 3.2. Primary outcomes analysis

In the univariate analysis of the MainEndpoint I group, significant differences were observed in Dage (*p* = 0.002), AST/ALT (*p* = 0.038), MELD (*p* = 0.005), MLR (*p* = 0.001), WIT (*p* = 0.002), Anhepatic (*p* = 0.001), Graft weight (*p* = 0.002), Neutrophils (*p* = 0.019), Total Cholesterol (*p* = 0.031), and Creatinine (*p* = 0.020). These variables were included in the univariate logistic regression, which identified Dage (OR: 1.035, *p* = 0.002, 95% CI: 1.012–1.058), Graft weight (OR: 2.338, *p* = 0.016, 95% CI: 1.170–4.669), Anhepatic (OR: 1.045, *p* < 0.001, 95% CI: 1.021–1.070), Neutrophils (OR: 1.189, *p* = 0.023, 95% CI: 1.024–1.380), Total Cholesterol (OR: 1.247, *p* = 0.039, 95% CI: 1.011–1.536), MLR (OR: 2.434, *p* = 0.003, 95% CI: 1.365–4.343), and WIT (OR: 1.218, *p* = 0.004, 95% CI: 1.066–1.391) as significant risk factors for postoperative complications requiring surgical or interventional treatment. Subsequent Wald stepwise logistic regression revealed Dage (OR: 1.026, *p* = 0.034, 95% CI: 1.002–1.050), MLR (OR: 1.973, *p* = 0.021, 95% CI: 1.106–3.519), Anhepatic (OR: 1.035, *p* = 0.006, 95% CI: 1.010–1.060), and Total Cholesterol (OR: 1.259, *p* = 0.031, 95% CI: 1.022–1.552) as independent risk factors of MainEndpoint I.

In the univariate analysis for MainEndpoint II, significant differences were observed between groups in total bilirubin (*p* = 0.001), CIT (*p* = 0.005), CAR (*p* = 0.004), INR (*p* = 0.045), ALBI (*p* = 0.019), lactate (*p* = 0.003), and SII (*p* = 0.001). These variables were subsequently included in univariate logistic regression, which identified significant associations with life-threatening complications after surgery: total bilirubin (OR = 1.003, *p* = 0.004, 95% CI: 1.001–1.005), CIT (OR = 1.010, *p* = 0.001, 95% CI: 1.004–1.016), CAR (OR = 2.623, *p* = 0.004, 95% CI: 1.363–5.049), ALBI (OR = 1.820, *p* = 0.018, 95% CI: 1.109–2.986), lactate (OR = 2.164, *p* = 0.006, 95% CI: 1.253–3.738), and SII (OR = 1.001, *p* = 0.004, 95% CI: 1.000–1.002). Stepwise backward logistic regression further identified CIT (OR = 1.008, *p* = 0.009, 95% CI: 1.002–1.014), CAR (OR = 2.255, *p* = 0.028, 95% CI: 1.094–4.649), lactate (OR = 2.078, *p* = 0.017, 95% CI: 1.140–3.790), and SII (OR = 1.001, *p* = 0.034, 95% CI: 1.000–1.002) as independent risk factors for MainEndpoint II. Results from the univariate and multivariate logistic regression analyses for both endpoints are presented in Tables 2 and 3.

**Table 2.** The univariate logistic regression results for the main endpoint.

	CD ≥ III and < IV(*n* = 101)		CD > IV(*n* = 39)	
Variables	Univariate OR (95% CI)	P-value	Univariate OR (95% CI)	P-value
Donor Age	1.035(1.012-1.058)	**0.002**	0.988(0.962-1.014)	0.368
CRP	0.984(0.968-1.000)	**0.046**	1.028(1.009-1.047)	**0.004**
CAR	0.574(0.325-1.016)	0.057	2.623(1.363-5.049)	**0.004**
Neutrophil	1.189(1.024-1.380)	**0.023**	0.936(0.773-1.134)	0.499
PLT	0.996(0.991-1.000)	**0.039**	1.009(1.004-1.014)	**<0.001**
SII	1.000(0.999-1.000)	0.267	1.001(1.001-1.002)	**0.004**
Monocyte	1.829(0.765-4.373)	0.175	2.923(1.132-7.551)	**0.027**
MLR	2.434(1.365-4.343)	**0.003**	1.100(0.595-2.034)	0.762
Total B	0.999(0.997-1.001)	0.171	1.003(1.001-1.005)	**0.004**
ALBI	0.806(0.557-1.167)	0.253	1.820(1.109-2.986)	**0.018**
ALT	0.999(0.998-1.000)	**0.045**	1.000(1.000-1.001)	0.307
TotalCholesterol	1.247(1.011-1.536)	**0.039**	0.898(0.690-1.169)	0.423
Lactate	0.782(0.522-1.169)	0.23	2.164(1.253-3.738)	**0.006**
GraftWeight	2.338(1.170-4.669)	**0.016**	0.844(0.359-1.984)	0.698
Anhepatic	1.045(1.021-1.070)	**<0.001**	0.993(0.971-1.017)	0.575
CIT	0.999(0.995-1.004)	0.817	1.010(1.004-1.016)	**0.001**
WIT	1.218(1.066-1.391)	**0.004**	1.074(0.914-1.261)	0.385

The results of univariate logistic regression analysis for two main endpoints, with a P-value less than 0.05 considered statistically significant. CAR: C-reactive protein to albumin ratio; NLR: Neutrophil to lymphocyte ratio; PLR: Platelet to lymphocyte ratio; SII: Systemic immune-inflammation index; MLR: Monocyte to lymphocyte ratio; ALBI: Albumin-bilirubin score; The logistic regression analysis was performed using the Bootstrap method with 1000 resampling iterations.

**Table 3.** The multivariate logistic regression results for the main outcome.

	CD ≥ III and < IV(*n* = 101)		CD > IV(*n* = 39)	
Variables	Multivariate OR (95% CI)	P-value	Multivariate OR (95% CI)	P-value
Donor Age	1.024(1.000-1.048)	**0.046**	N.A.	N.A.
MELD Scores	0.974(0.928-1.022)	0.282	1.004(0.942-1.071)	0.425
CAR	N.A.	N.A.	2.255(1.094-4.649)	**0.028**
Neutrophil	1.100(0.933-1.298)	0.257	N.A.	N.A.
SII	N.A.	N.A.	1.001(1.000-1.002)	**0.034**
MLR	1.953(1.092-3.495)	**0.024**	N.A.	N.A.
Total B	N.A.	N.A.	1.002(1.000-1.004)	0.094
ALBI			1.265(0.647-2.471)	0.492
AST/ALT	0.926(0.719-1.193)	0.551	N.A.	N.A.
TotalCholesterol	1.248(1.009-1.543)	**0.041**		
INR	N.A.	N.A.	1.099(0.723-1.671)	0.657
Lactate			2.078(1.140-3.790)	**0.017**
GRWR	1.825(0.881-3.777)	0.105	N.A.	N.A.
Anhepatic	1.040(1.016-1.066)	**0.001**		
CIT	N.A.	N.A.	1.008(1.002-1.014)	**0.009**
WIT	1.069(0.912-1.253)	0.412	N.A.	N.A.

The results of multivariate logistic regression analysis for two main endpoints, with a P-value < 0.05 considered statistically significant; The logistic regression analysis was performed using the Bootstrap method with 1000 resampling iterations. N.A. = Non applicable.

### 3.3. Secondary outcomes analysis

**Figure 2.**
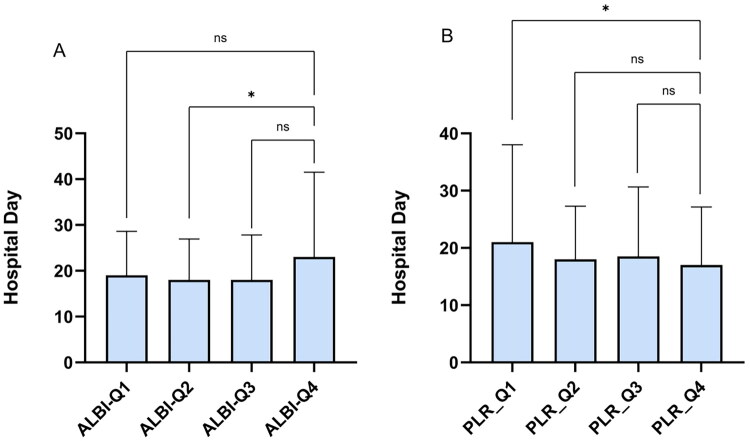
Quartile group analysis of total hospital stay based on ALBI and PLR. Quartile group analysis of total hospital stay based on ALBI; (A) Quartile group analysis of total hospital stay based on ALBI. (B) Quartile group analysis of total hospital stay based on PLR; Using the Dunn test and Kruskal-Wallis test for inter-group significance comparison. Significant differences between groups are indicated by *, * represents p < 0.05, indicating statistical significance; ** represents p < 0.01, indicating strong statistical significance; *** represents p < 0.001, indicating very strong statistical significance; NS = No significance.

Considering potential redundancy, collinearity analysis was performed on all composite indicators (Table S1). Due to strong collinearity, CRP and PLT were excluded from EAD analysis. Univariate and multivariate logistic regressions showed that NLR was not significantly associated with outcomes. CAR was an independent risk factor for postoperative EAD (OR: 2.484, *p* = 0.001, 95% CI: 2.562–11.737) with an optimal cut-off of 0.45 by ROC. Patients with CAR ≥ 0.45 exhibited a significantly increased incidence of EAD compared to those with CAR < 0.45 (*p* < 0.001), which is consistent with findings by Jaesik Park et al. [30] For 90-day graft loss, only CAR differed significantly between groups (*p* = 0.038), but logistic regression with 1000 Bootstrap iterations showed no composite indicators as independent risk factors. Spearman correlation analysis showed that total hospital stay was weakly positively correlated with ALBI (*r* = 0.136, *p* = 0.048) and weakly negatively correlated with PLR (r = −0.178, *p* = 0.01). Grouping by CAR cut-off (Table S2) showed longer ICU stays in the high CAR group (median 2.57 [0–4] days) vs. low CAR group (1.08 [0] days, *p* < 0.001), with CAR positively correlated with ICU length (*r* = 0.239, *p* < 0.001). In 90-day postoperative CCI analysis, SII was an independent risk factor for CCI > 75. ALBI (*r* = 0.173, *p* = 0.012), MLR (*r* = 0.246, *p* < 0.001), and SII (*r* = 0.196, *p* = 0.004) all positively correlated with CCI by Spearman analysis. ALBI and PLR were grouped by quartiles and plotted in bar charts to explore their relationship with total hospital stay and group differences (Figure 2A,B).

### 3.4. Nomogram performance and clinical benefit analysis

Based on the results of the main outcomes, we created a Nomogram (Figure S1A,B) and plotted the ROC curve using the Bootstrap method (Figure S2A,B). The mean AUC for Nomogram A was 0.735, and the mean AUC for Nomogram B was 0.792, both showing good predictive capability for outcomes. Next, we plotted the calibration curves (Figure S3A,B), where the mean absolute error (MAE) for Nomogram A was 0.014 and for Nomogram B was 0.300. The Hosmer-Lemeshow (HL) test for Nomogram A showed *p* = 0.757, and for Nomogram B, *p* = 0.708. The curves show good alignment with the ideal curve, indicating stable calibration ability. We then plotted the Decision Curve Analysis (DCA) curve (Figure S4A,B), which showed that both Nomogram A and B provided significant decision-making benefits at multiple threshold probabilities compared to the all-intervention and no-intervention curves, further confirming their clinical application potential. Additionally, the Clinical Impact Curve (CIC) analysis (Figure S5A,B) showed that the model could accurately predict the actual number of positive cases at different risk thresholds, demonstrating strong clinical predictive ability.

## 4. Discussion

Since their initial investigation, inflammation-immune composite markers have been widely studied and shown to correlate with outcomes across various patient populations, including those with critical illness [25,38], liver cirrhosis awaiting LT [39,40], 1-year mortality after LDLT [31,32], and tumor progression, such as disease-free survival, advanced staging [34,41,42] and postoperative recurrence [24,33,42–45]. However, in the context of LT, existing studies mainly focus on LDLT or isolated postoperative indicator [46].

Iakovos et al. [4]reported that CAR was not associated with EAD under the Olthoff definition, though the high CAR group had significantly higher MEAF scores. In contrast, our study identified CAR as an independent risk factor for EAD with an optimal cut-off of 0.45. Grouping by this value showed significantly higher EAD incidence in the high CAR group (*p* < 0.001), consistent with Park et al. [31] Consistent with previous studies [47]. SII reached statistical significance in multivariate analysis (OR = 1.001, *p* = 0.049, 95% CI: 1.000–1.002), but the narrow CI indicates minimal clinical relevance, leading to its exclusion. Although CAR was not an independent predictor for 90-day graft loss, its incidence was higher in the high CAR group (7.44% vs 3.41%).

**Figure 3.**
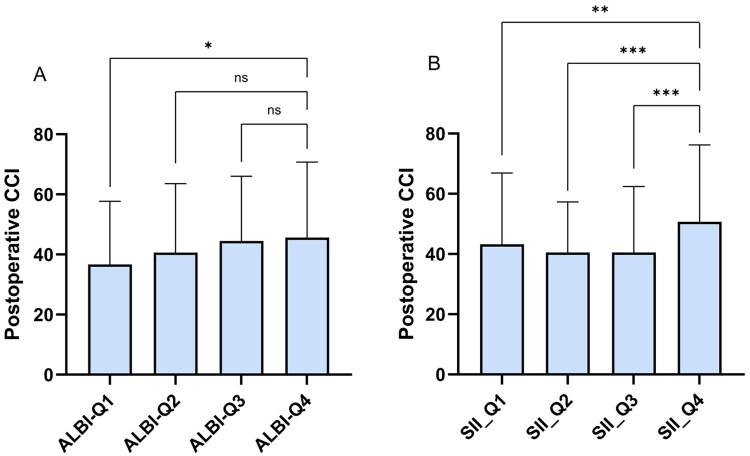
Quartile group analysis of CCI based on ALBI and SII. (A) Quartile group analysis of CCI based on ALBI; (B) Quartile group analysis of CCI based on SII; Using the Dunn test and Kruskal-Wallis test for inter-group significance comparison. Significant differences between groups are indicated by *represents p < 0.05, indicating statistical significance; **represents p < 0.01, indicating strong statistical significance; ***represents p < 0.001, indicating very strong statistical significance; NS = No significance.

In Spearman correlation analysis, ALBI and PLR showed weak correlations with total hospital stay, with the fourth quartiles of ALBI and PLR exhibiting significantly longer and shorter stays, respectively (Figure 2A,B). ALBI, initially developed to assess liver function in HCC patients [48], has demonstrated utility across liver malignancies including intrahepatic cholangiocarcinoma [49–51]. A negative correlation between ALBI and PLR was confirmed (*p* < 0.001, r=-0.260), while ALBI showed no association with lymphocyte counts (*p* = 0.807, *r* = 0.017), suggesting better preoperative liver function may predict improved postoperative outcomes. PLR has also been reported as an independent risk factor for mortality and postoperative recurrence in liver transplant patients with liver cancer [31,34,41,52]. Higher CAR was associated with prolonged ICU stay; mean ICU durations in high CAR and high SII groups (Table S3) were approximately 137% and 144% greater than in low groups, respectively. CAR poorly predicted 90-day mortality (*p* = 0.430), consistent with Iakovos et al. Multivariate analysis identified MLR as an independent risk factor for 90-day mortality (OR: 2.261, *p* = 0.028, 95% CI: 1.093-4.681), correlating with increased postoperative CCI (*r* = 0.246, *p* < 0.001). No composite markers independently predicted CCI >50; however, SII was an independent risk factor for CCI >75. ALBI was also correlated with an increase in CCI (*r* = 0.173, *p* = 0.012), and significant differences in CCI were observed among the quartile groups of both ALBI and SII (Figure 3A,B). The high SII group incurred approximately 18.9% greater total costs (*p* < 0.001), supported by Spearman and quartile analyses showing higher hospitalization costs in the top SII quartile (*p* < 0.001, *r* = 0.228). Detailed logistic regression results for binary outcomes are in Table S4.

In terms of main outcomes, logistic regression showed that MLR effectively predicted complications requiring surgical or interventional intervention (CD ≥ III but < IV) (AUC = 0.674, cut-off = 0.57). CAR (AUC = 0.647) and SII (AUC = 0.668, cut-off = 418.73) also showed good predictive value for severe complications (CD ≥ IV), and all were independent risk factors. These results suggest that SII and MLR are useful for identifying early severe complications (CD ≥ IV) and those requiring intervention (CD ≥ III but < IV), respectively. In terms of CCI, they also reflect the risk of multiple complications, which often leads to increased postoperative interventions and higher hospitalization costs. Preoperative SII, calculated based on neutrophil, platelet, and lymphocyte counts, serves as a comprehensive indicator reflecting the systemic inflammatory burden of liver transplant recipients and is influenced by multiple factors. From a cellular and molecular perspective, patients with end-stage liver disease often present with immune suppression characterized by decreased lymphocyte counts and elevated peripheral neutrophil levels. The latter migrate to the liver through CXCR1/2-mediated pathways [53]. After migrating into the liver parenchyma, neutrophils contribute directly to hepatocyte necrosis and apoptosis through degranulation, reactive oxygen species (ROS) generation, and neutrophil extracellular traps (NETs) formation, thereby activating signaling pathways such as caspase and MAPK/JAK-STAT [54,55]. Necrotic or apoptotic hepatocytes subsequently release DAMPs including HMGB1, ATP, and DNA fragments. These DAMPs further activate Kupffer cells and neutrophils in the liver through TLR4/RAGE receptors or stimulate macrophages and dendritic cells *via* TLR9 receptors, promoting the release of proinflammatory cytokines such as IL-1β [56]. Activated Kupffer cells then amplify the inflammatory response through the ROS-NLRP3-caspase-1-IL-1βpathway and secretion of proinflammatory factors like TNF-α [57,58]. Ultimately, a sterile inflammation amplification loop centered on neutrophils is established. This local dysregulated inflammatory immune microenvironment reflects increased peripheral immune burden preoperatively. In recipients with significantly elevated preoperative SII, neutrophil effector functions may already be in a preactivated state, enabling rapid response to DAMPs signals during reperfusion in liver transplantation, resulting in exacerbated sterile inflammation and IRI, and thereby increasing the risk of severe postoperative complications. Interestingly, when MLR was stratified by cut-off (Table S5), the high MLR group had a significantly shorter ICU stay than the low group (*p* = 0.038). Although Spearman analysis did not show a significant correlation (r=–0.084, *p* = 0.224), this, together with main outcome analysis, indicates that MLR may better reflect multiple less-severe complications compared to SII, which tend to not require intensive care. Similar to SII, CAR showed good predictive value for severe complications (CD ≥ IV), prolonged ICU stay, and EAD. However, it was not significantly associated with CCI in either Spearman or logistic regression, suggesting limited predictive value for the burden of multiple complex complications, consistent with findings from Iakovos et al. [4]. NLR was not significantly associated with any outcome. The Nomogram developed for both main outcomes showed good accuracy and clinical benefit across multiple evaluations (calibration curve, DCA, CIC), offering a practical tool for postoperative complication risk assessment.

Studies have confirmed that severe IRI during LT causes significant postoperative changes in lab results [59], especially CRP and ALB, affected by liver dysfunction and stress [26,30,60]. From the perspective of predictive modeling, many complications arise early and progress quickly, so relying on postoperative parameters to predict risk may lack timeliness.

It must be acknowledged that our study has certain limitations: first, its retrospective and single-center design limits the ability to eliminate preoperative recipient biases. Second, all laboratory indicators were cross-sectional, based on a single time point, making it difficult to assess longitudinal changes (improvement or deterioration). Future studies could explore how such dynamic changes influence postoperative outcomes. Third, inflammation-immune composite indicators lack specificity and may be affected by differences in patient populations, treatment strategies, and laboratory methods. Finally, the model was only internally validated using the Bootstrap method and has not been tested on external datasets, underscoring the need for further validation in large, multi-center cohorts.

Although molecular biomarkers offer high specificity and sensitivity as predictors [61,62], the value of composite indicators lies in their simplicity and accessibility. Their molecular mechanisms have yet to be fully elucidated, warranting further cellular and molecular research. To our knowledge, no prior study has systematically examined the predictive value of common inflammation-immune composite indicators in DDLT. Our study fills this gap by developing a Nomogram based on CD classification, aiming to provide a practical risk assessment tool for clinicians and guidance for future predictive model research.

## Supplementary Material

Supplemental Material

## Data Availability

The original dataset for this study cannot be publicly shared due to legal restrictions. However, de-identified versions of the original dataset are available upon request from the corresponding author.
