# Supplementary material for: The evaluation of inflammatory and immune composite markers for complications after deceased donor liver transplantation – a retrospective cohort study
Source: Ann Med. 2025 Jul 24;57(1):2536757. doi: 10.1080/07853890.2025.2536757 (PMC12291190; doi:10.1080/07853890.2025.2536757)
Supplement: Supplemental Material [file IANN_A_2536757_SM1435.zip › suppl_data/Figures.docx]

Supplement Figure 1Nomogram of MainEndpointⅠand Ⅱ

Supplement Figure 1A: Nomogram of MainEndpoint I；Supplement Figure 1B: Nomogram of MainEndpoint II；The SII was log-transformed in the model due to the large values of the variable.

Supplement Figure 2 ROC curves of Nomogram A and B based on 1000 bootstraps

Supplement Figure 2A: ROC curve of Nomogram A based on 1000 bootstrap resamples；The blue line represents the mean ROC curve. Mean AUC = 0.735；Supplement Figure 2B: ROC curve of Nomogram B based on 1000 bootstrap resamples；The blue line represents the mean ROC curve；Mean AUC = 0.792；Gray area: ROC curve results from 1000 bootstrap resamples of the model

Supplement Figure 3 Calibration curves of Nomogram A and B

Supplement Figure 3A: Calibration curve of Nomogram A, Mean absolute error (MAE) = 0.014；Supplement Figure 3B: Calibration curve of Nomogram B, Mean absolute error (MAE) = 0.03；Ideal Line: Represents perfect calibration, where predictions exactly match the actual values.Bias-corrected Line: The line obtained after correcting the model.Apparent Line: Represents the model's performance before any correction.

Supplement Figure 4 Decision curve analysis of Nomogram A and B

Supplement Figure 4A: DCA analysis of Nomogram A;Supplement Figure 4B: DCA analysis of Nomogram B;Red line: Benefit curve for applying intervention to all samples;Green line: Benefit curve for applying no intervention to all samples;Blue line: Benefit curve for selectively applying intervention based on the model to all samples.Both analyses show that the application of the model within the risk threshold yields higher benefits than full intervention.

Supplement Figure 5 Clinical Impact Curve analysis of Nomogram A and B

Supplement Figure 5A: CIC analysis of Nomogram A;Supplement Figure 5B: CIC analysis of Nomogram B;Red line: Predicted number of positive samples by the model;Blue line: Actual number of positive samples.Both analyses show that within the risk threshold, the predicted number of positive samples by the model closely matches the actual number of positive samples.
