# Supplementary material for: The evaluation of inflammatory and immune composite markers for complications after deceased donor liver transplantation – a retrospective cohort study
Source: Ann Med. 2025 Jul 24;57(1):2536757. doi: 10.1080/07853890.2025.2536757 (PMC12291190; doi:10.1080/07853890.2025.2536757)
Supplement: Supplemental Material [file IANN_A_2536757_SM1435.zip › suppl_data/Supplementary_Table_legends.docx]

**Supplementary Table legends**

Supplementary Table 1.Collinearity diagnostics of inflammatory-immune markers.

CRP:C-reactive protein;CAR: C-reactive protein to albumin ratio; NLR: Neutrophil to lymphocyte ratio; PLR: Platelet to lymphocyte ratio; SII: Systemic immune-inflammation index; MLR: Monocyte to lymphocyte ratio; ALBI: Albumin-bilirubin score;VIF: Variance Inflation Factor; values >5 suggest moderate, and >10 indicate severe multicollinearity.

Supplementary Table 2.Comparison of parameters based on the optimal CAR cutoff(0.45).

MELD: Model for End-Stage Liver Disease;CRP:C-reactive protein;CAR: C-reactive protein to albumin ratio; NLR: Neutrophil to lymphocyte ratio; PLR: Platelet to lymphocyte ratio; SII: Systemic immune-inflammation index; MLR: Monocyte to lymphocyte ratio; ALBI: Albumin-bilirubin score;GRWR:Graft-to-recipient weight ratio;CCI: Comprehensive Complication Index, a continuous scale that quantifies the overall burden of postoperative complications.EAD:Early allograft dysfunction;CD:Clavien-Dindo classification of surgical complications.

Supplementary Table 3.Comparison of parameters based on the optimal SII cutoff(418.73).

MELD: Model for End-Stage Liver Disease;CRP:C-reactive protein;CAR: C-reactive protein to albumin ratio; NLR: Neutrophil to lymphocyte ratio; PLR: Platelet to lymphocyte ratio; SII: Systemic immune-inflammation index; MLR: Monocyte to lymphocyte ratio; ALBI: Albumin-bilirubin score;GRWR:Graft-to-recipient weight ratio;CCI: Comprehensive Complication Index, a continuous scale that quantifies the overall burden of postoperative complications.EAD:Early allograft dysfunction;CD:Clavien-Dindo classification of surgical complications.

Supplementary Table 4.Performance of inflammatory-immune composite markers in binary outcomes.

CAR: C-reactive protein to albumin ratio;NLR: Neutrophil to lymphocyte ratio; PLR: Platelet to lymphocyte ratio; SII: Systemic immune-inflammation index; MLR: Monocyte to lymphocyte ratio; ALBI: Albumin-bilirubin score.

Supplementary Table 5.Comparison of parameters based on the optimal MLR cutoff(0.57).

MELD: Model for End-Stage Liver Disease;CRP:C-reactive protein;CAR: C-reactive protein to albumin ratio; NLR: Neutrophil to lymphocyte ratio; PLR: Platelet to lymphocyte ratio; SII: Systemic immune-inflammation index; MLR: Monocyte to lymphocyte ratio; ALBI: Albumin-bilirubin score;GRWR:Graft-to-recipient weight ratio;CCI: Comprehensive Complication Index, a continuous scale that quantifies the overall burden of postoperative complications.EAD:Early allograft dysfunction;CD:Clavien-Dindo classification of surgical complications.
