# Supplementary figures and images for: The evaluation of inflammatory and immune composite markers for complications after deceased donor liver transplantation – a retrospective cohort study
Source: Ann Med. 2025 Jul 24;57(1):2536757. doi: 10.1080/07853890.2025.2536757 (PMC12291190; doi:10.1080/07853890.2025.2536757)

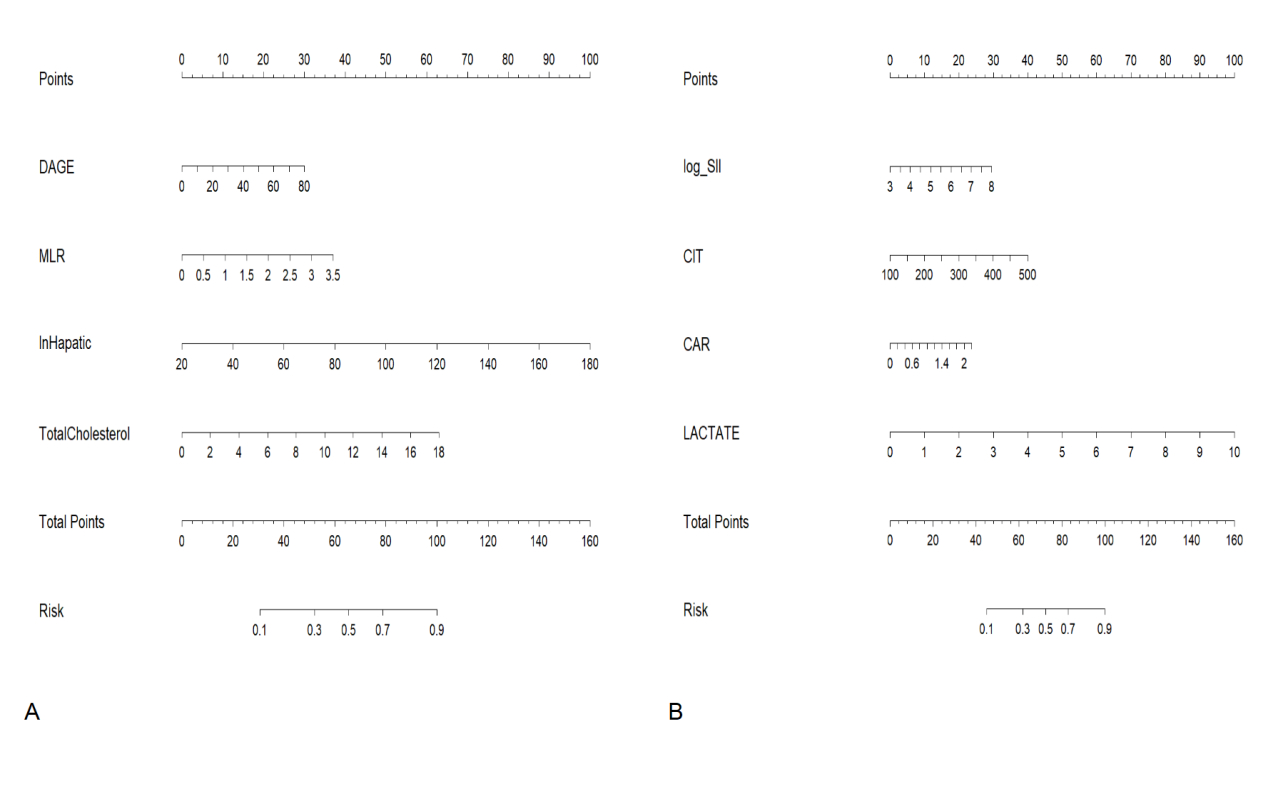

Supplement: Supplemental Material [file IANN_A_2536757_SM1435.zip › suppl_data/Supplement Figure 1 Revised.jpg]

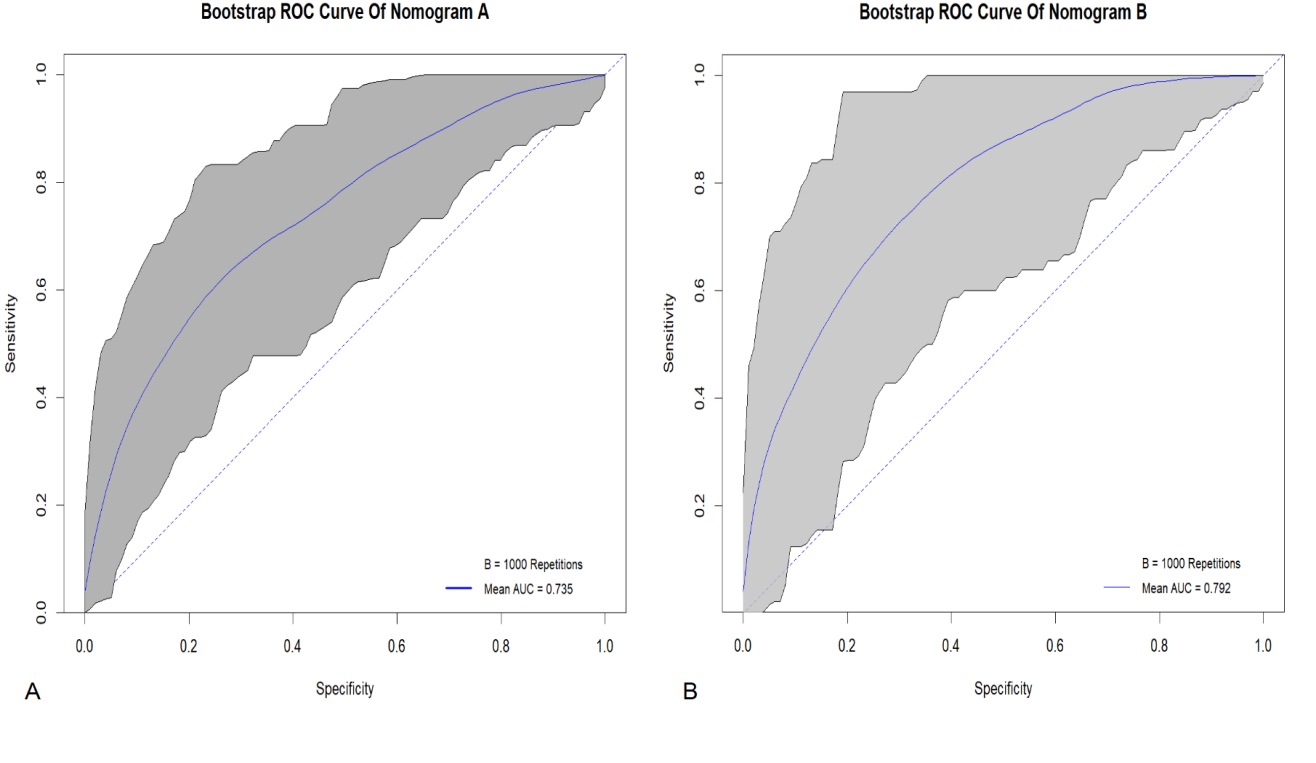

Supplement: Supplemental Material [file IANN_A_2536757_SM1435.zip › suppl_data/Supplement Figure 2 Revised.jpg]

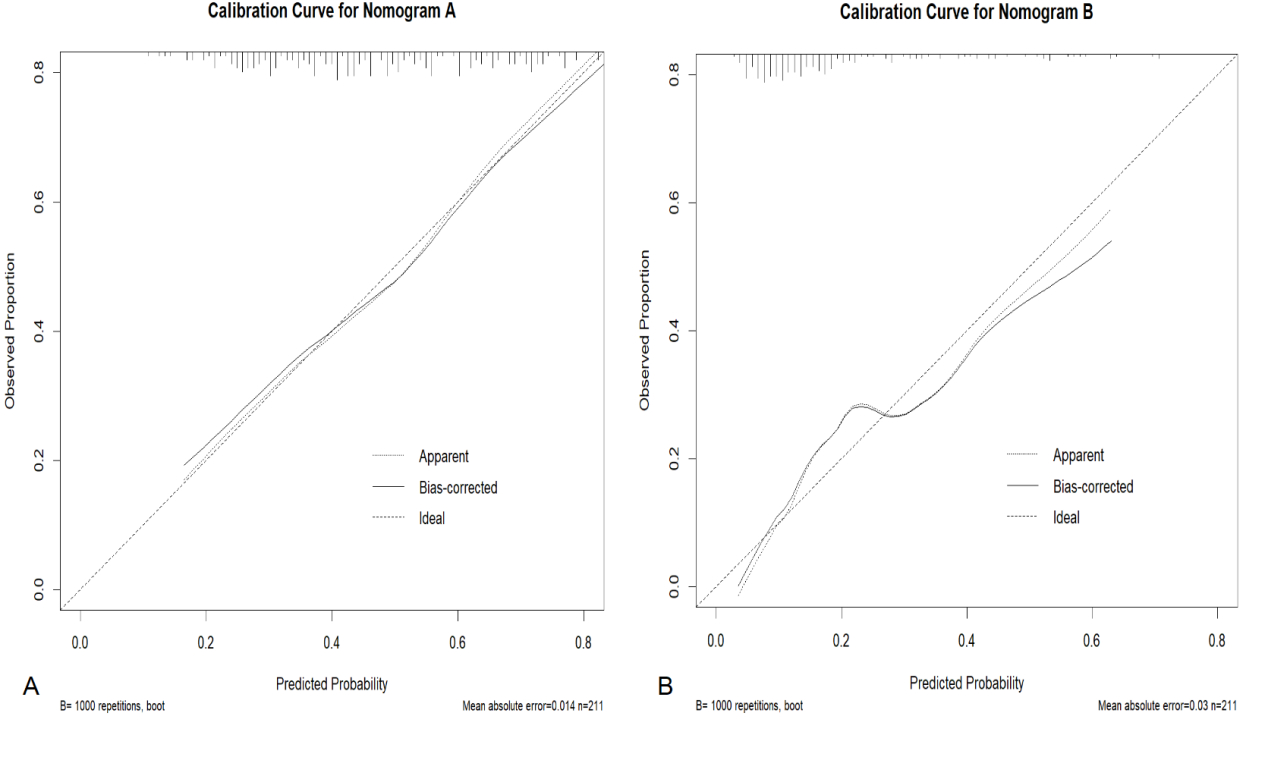

Supplement: Supplemental Material [file IANN_A_2536757_SM1435.zip › suppl_data/Supplement Figure 3 Revised.jpg]

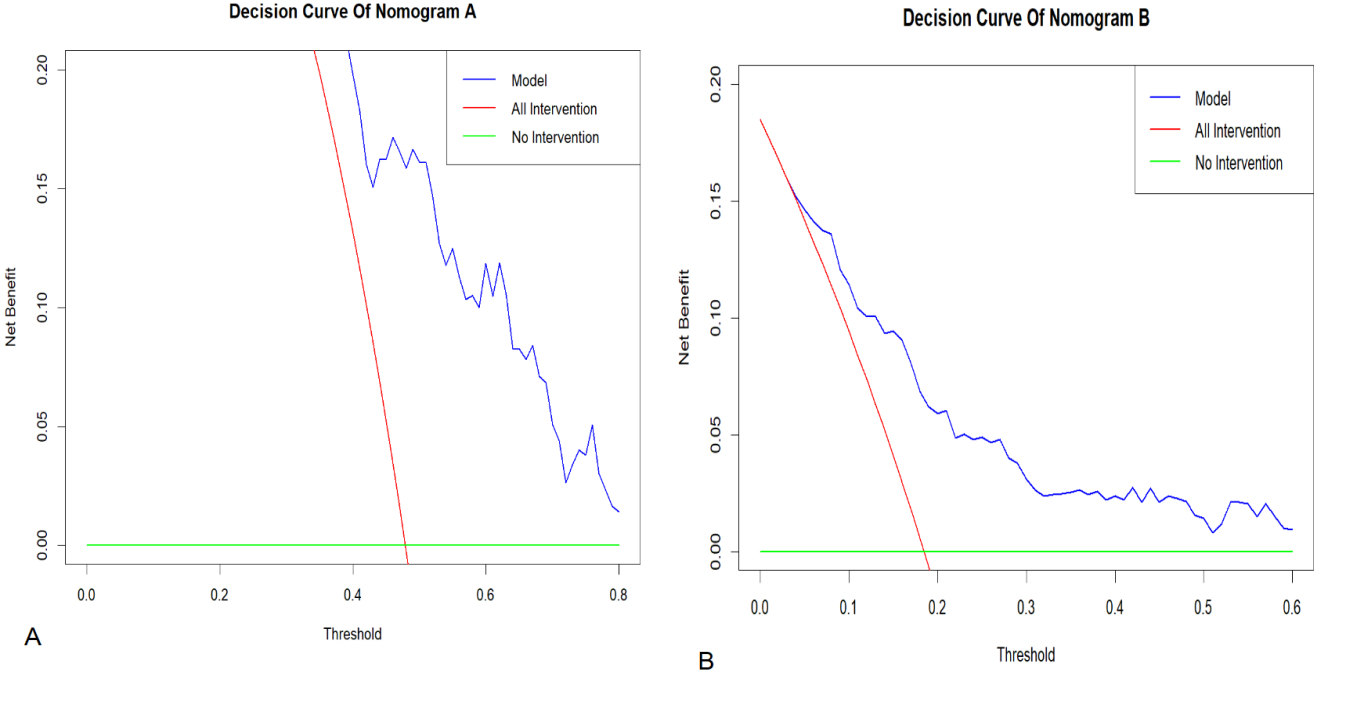

Supplement: Supplemental Material [file IANN_A_2536757_SM1435.zip › suppl_data/Supplement Figure 4 Revised.jpg]

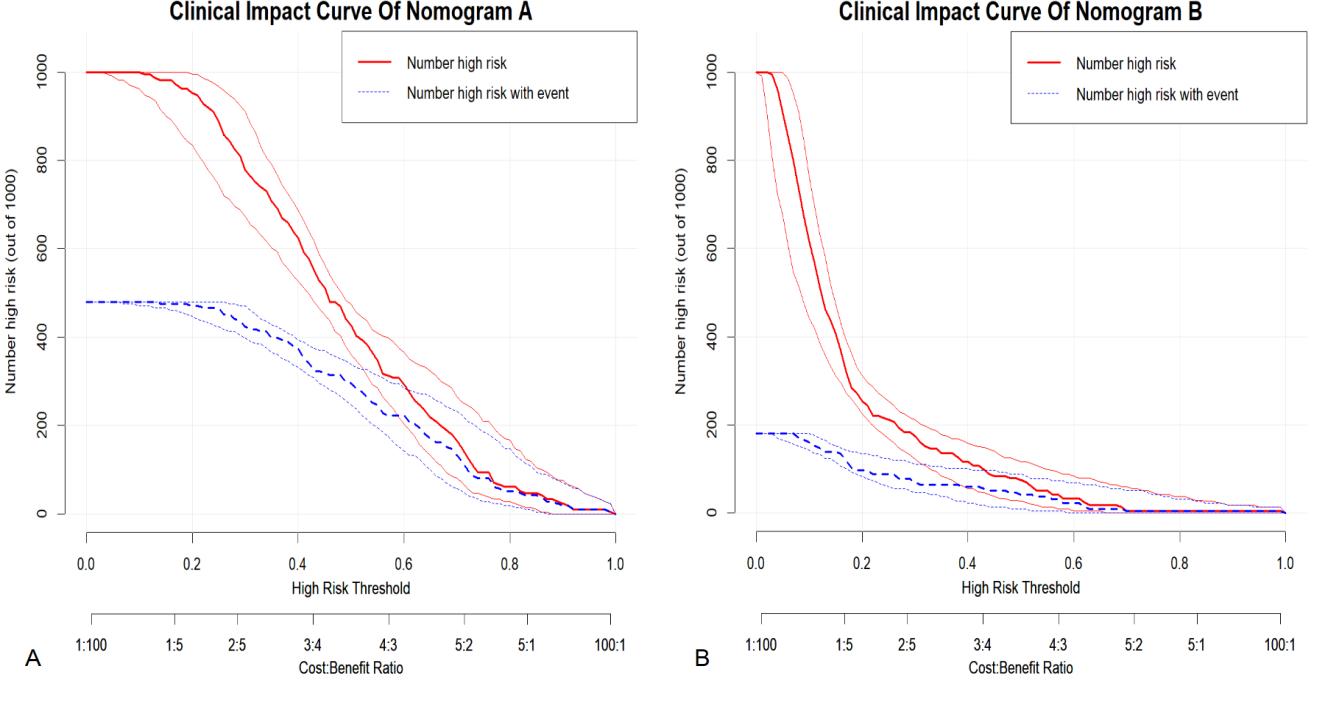

Supplement: Supplemental Material [file IANN_A_2536757_SM1435.zip › suppl_data/Supplement Figure 5 Revised.jpg]
